# Adapting CBT for youth anxiety: Flexibility, within fidelity, in different settings

**DOI:** 10.3389/fpsyt.2023.1067047

**Published:** 2023-03-01

**Authors:** Philip C. Kendall, Julia S. Ney, Colleen A. Maxwell, Kyler R. Lehrbach, Rafaella J. Jakubovic, Dominique S. McKnight, Abbey L. Friedman

**Affiliations:** Department of Psychology and Neuroscience, Child and Adolescent Anxiety Disorders Clinic, Temple University, Philadelphia, PA, United States

**Keywords:** cognitive behavioral therapy (CBT), anxiety, youth, flexibility, adaptation

## Abstract

Anxiety disorders are common in youth, associated with impairments in daily functioning, and often persist into adulthood when untreated. Cognitive behavioral therapy (CBT) for youth anxiety is a well-established intervention and has been modified to fit several treatment settings. Despite decades of results supporting the efficacy of CBT, there is a large gap in access to this treatment and a need to consider how it can best be administered flexibly to increase uptake and personalization. We first discuss the core components of treatment for CBT through the lens of the Coping Cat treatment. Next, we review the empirical findings regarding adjustments made for CBT for youth anxiety delivered (a) in schools, (b) in community settings, (c) through telehealth, (d) through online computer programs, and (e) by caregivers at home. In each setting, we provide specific suggestions for how to implement CBT with flexibility while maintaining fidelity.

## 1. Introduction

Anxiety disorders are the most common mental health problem for children and adolescents and have been found to be present in nearly 20% of youth (1). Youth anxiety disorders are associated with impairments in daily functioning and typically persist into adulthood when they are associated with the onset of comorbid mental health disorders (2, 3). Cognitive behavioral therapy (CBT) for youth anxiety is a well-established intervention, with meta-analyses concluding that approximately 60% of youth recover following treatment (4). In the past decade, CBT for youth anxiety has been adapted to diverse settings, including schools, community mental health centers, online programs, and telehealth, and has been administered through parent training. CBT for youth anxiety, within an upbeat therapeutic relationship, targets maladaptive anxious thinking to foster adaptive information processing, reduce anxious feelings, and enhance approach behavior (reduce avoidance) using rapport building, psychoeducation, cognitive restructuring, problem-solving, and exposure tasks (with rewards). CBT for youth anxiety can be accomplished using several empirically supported programs, one of which is the *Coping Cat*. Consistent with reviewer input, we are keeping the report tight with sole reference to the *Coping Cat*. That said, our suggestions are applicable to CBT in general. The standard protocol for this intervention includes 16 50-min sessions, which cover several key components. We first overview these key components of youth anxiety treatments that may be flexibly adapted and next discuss changes that may be made in different settings to apply CBT for youth anxiety flexibly and with fidelity.

## 2. Key components of youth anxiety treatments

There are treatment manuals that prescribe, with detail, the guidelines for delivering an evidence-based intervention for youth anxiety, and although there is some variability among them, several key components are consistent across those with demonstrated efficacy. Manuals and personalized approaches differ in the specific ways that the intervention is delivered (e.g., interactive activity, psychoeducational game, worksheet), often referred to as the form of the intervention, while the function, or the purpose of a given form is largely consistent across manuals. We have identified key components of evidence-based approaches to treatments for youth anxiety by their function, describe their empirical basis, and provide examples of different forms that they may take to maintain fidelity and be successful.

### 2.1. Building rapport

Therapeutic alliance has been positively, though modestly, associated with treatment outcomes for youth receiving treatment for anxiety (5, 6). Meta-analytic work has identified effect size estimates as 0.10–0.14. The results suggest that the importance of the therapeutic relationship may be stronger in the case of individual interventions, youth with mixed problem presentations, younger children, and treatment-seeking samples (7, 8). Importantly, findings also suggest that the role of the alliance was greater when measured at the beginning of treatment, suggesting that it may be important to start strong with anxious youth (7, 8). Rapport building can take numerous forms. Many youth find comfort easing into the therapeutic process by playing “get to know you” games, while older youth may prefer to simply chat. These practices can be extended throughout therapy by setting aside a few minutes at the end of a session to play a game (not necessarily therapy-related) or watch a YouTube video, with the goal that the client leaves the session feeling positive and connected. This functions as a reward and can reinforce the youth’s engagement in treatment. CBT uses rewards and praise for desired behaviors in therapy and at home. An important feature of youth therapy is the fact that the decision to attend therapy is not solely made by the youth; rather, many youth present to therapy as a result of a caregiver’s desire for treatment. As such, as part of building rapport, it can be useful for therapists to inquire about the youth’s motivation for treatment and validate any ambivalence.

### 2.2. Psychoeducation about anxiety

Psychoeducation has been identified as a core component of CBT broadly (9). In the context of youth anxiety, it has been found that the extent to which youth engage in psychoeducational components of treatment positively predicts treatment outcomes (10). The function of psychoeducation is to increase the youth’s knowledge about anxiety, including what it feels/looks like, where it comes from, how it works, how we treat it and dispelling myths about anxiety. In practice, therapists may address the causes of anxiety by teaching the adaptive nature of anxiety. Therapists can normalize day-to-day experiences of anxiety by sharing their own examples, pointing out with the youth’s help different ways that anxiety can be helpful (e.g., stops us from running into traffic), and then discussing ways in which anxiety that gets too high can become unhelpful (e.g., so stressed for a test you can’t study). This conversation allows the therapist to set expectations for treatment: the goal of therapy is not to eliminate anxiety but to be able to cope with anxiety without letting it get in the way. To teach how anxiety works, the cognitive triangle is introduced, with particular emphasis on the connection between the emotion of anxiety and the physical symptoms it produces. Activities can be used to identify the specific physical symptoms that the youth experiences; for example, younger children may enjoy tracing their body and drawing the manifestations of their physical symptoms (e.g., butterflies in the stomach, sweaty hands), while older youth may prefer to use a list of common symptoms to identify which symptoms they feel and at what levels of anxiety they begin to experience them (e.g., shaky hands, heart racing).

### 2.3. Cognitive restructuring

Consistent with theory regarding the connection between feelings and thoughts, research on CBT for youth anxiety suggests that the cognitive restructuring component of treatment represents an active feature of change (11). A youth’s level of cognitive development is a factor that determines the relative utility of this component where older youth may derive greater benefit from mastery of these skills, and the benefits may be less for children under the age of ten or who have not yet developed meta-cognition (12). In practice, this process can be taught in three steps: (1) Catch it, (2) Check it, and (3) Change it. The Catch it step refers to the identification of thoughts, particularly when anxious physical symptoms have been detected. Youth who have difficulty distinguishing thoughts from feelings may benefit from filling in thought bubbles above drawings of themselves or others in various anxiety-provoking situations. For younger children, it may be helpful to externalize the idea of thoughts by identifying a character, such as a “worry monster” or a “guard dog,” who may be responsible for these anxious messages. Next, the Check it step involves examining whether the thought may be a cognitive distortion. Youth often benefit from a list of common thinking traps to compare with their thoughts. Youth may want to check if the thought is helpful (versus interfering). If the youth determines that the thought is not helpful or that they are falling into a thinking trap, the third step, Change it, provides an opportunity to respond to that thought with more helpful thoughts. For younger children, this may be framed as arguing back to the worry monster. For older youth, it is important that this step is not simply generating the opposite thought or telling themselves something they do not believe (e.g., I’m going to ace the test, not fail it). Rather, the function of this exercise is to achieve some level of cognitive flexibility by generating more balanced, believable thoughts (e.g., I may get a B or a C on the test but probably won’t fail it).

### 2.4. Exposure tasks

Conducting exposure tasks is considered the most critical component of CBT for youth anxiety (13, 14). In this context, exposures typically follow a graded approach in which youth, together with their caregivers and therapist, identify feared situations, rank them in terms of difficulty, begin with the easiest situation, and gradually move up the “fear hierarchy” as they progress through treatment. Recent meta-analytic work has demonstrated that across 75 studies of CBT with anxious youth, effect sizes were positively associated with the amount of in-session exposure (15). The field’s understanding of the mechanisms through which exposures affect change continues to advance, with recent findings suggesting that inhibitory learning models can be used to maximize their effectiveness (16). Specifically, this can involve emphasizing the violation of expectancies that occurs in exposure, deepening extinction by conducting exposures to distinct aspects of the feared situation first individually and then simultaneously, removing safety signals from the exposure experience, varying exposure content from week to week and across contexts, and developing retrieval cues to remind them of what they learned in exposures (e.g., holding the same paper clip in exposure practices as in real-life school presentations) (17). In practice, successful exposures can take many forms. Yet, prior to an exposure task, it is important that youth understand the reason for exposures or “challenges.” Metaphors like slowly entering a cold swimming pool can be used to describe the progressive and graded nature of exposure. Additionally, trust between the therapist and the client is important in preparation for this component of treatment. Youth should be positioned “in the driver’s seat” with assurance that they will be asked to do things they do not want to do, but they will not be forced to do anything they are not yet ready to do. In planning exposure tasks, a hierarchy of feared situations with increasing difficulty is created. To develop a hierarchy collaboratively, the therapist and youth (and can include the help of caregivers) set target behaviors that serve as the goals at the top of the hierarchy. The therapist may generate many scenarios that could approximate steps to the identified goal. The youth rank the scenarios and rate their perceived difficulty. The therapist can gain information about how to optimally design exposures by asking follow-up questions throughout this process (e.g., “Would it be easier or harder if…”). Of note, hierarchies are flexible, living documents that are adjusted as treatment progresses. Often, the first exposure identified by the youth is relatively “easy,” which may allow the youth to establish a sense of efficacy and agency when approaching exposure tasks. However, as youth progress through treatment, exposures on the hierarchy are scaffolded or adjusted so that they target moderate and increasing levels of anxiety.

When it comes time to engage in a feared situation, it is important firstly that the youth has agreed to do the task. It is helpful to set a specific and attainable goal for the exposure (e.g., “Talk to the stranger without leaving the room before the 4-minute mark” and not “Do a good job talking to a stranger”). Lastly, it is helpful to ask the youth what they expect to happen in the task so that this can be re-examined afterward, akin to a “behavioral experiment.” Throughout an exposure, the therapist tracks the youth’s distress through the use of a predetermined scale (e.g., 1–10); this information informs any adjustments that may be necessary for the therapist to make. For example, if the youth reports feeling so highly distressed that they anticipate escaping, it may be helpful to make the exposure slightly easier so as to allow the youth to meet the goal (back up but not back down). In the case that it becomes clear that the exposure is too difficult for the child, the therapist can work with the child to adjust the goal mid-exposure (e.g., “Rather than stay for 4 minutes, can you stay for 2?”). This adjustment provides an alternative response to anxious feelings that is not immediate escape, facilitating experiential learning that counters avoidance responses to anxiety. Following an exposure, youth are given an opportunity to assess their efforts, their performance in relation to the objective goal, and the accuracy of their expectations. The exposure task typically provides evidence that the youth was able to cope (e.g., achieve the objective goal) and that the anticipated catastrophe did not occur, violating their expectancy. Therapists provide ample praise, specifically focused on the effort put forth, rather than on a judgment of the outcome.

## 3. It’s all about making adjustments…within fidelity

Psychoeducation (e.g., normalizing anxiety), cognitive restructuring (changing anxious self-talk), and exposure tasks (e.g., behavioral disconfirmations of anticipated catastrophes) are central features of effective CBT, but they can and are implemented in varying ways. Many forms of CBT for youth anxiety include these core components. These treatments have been studied through randomized controlled trials—and they can be implemented flexibly and with complete fidelity outside the research studies. Attention to implementation in other settings (e.g., schools, online, community mental health centers) is particularly important as we consider how to best serve historically underserved populations of youth (e.g., youth living in low-income or rural settings, youth with comorbid mental health or neurodevelopmental concerns) who may be less likely to access specialty clinics. The phrase “flexibility within fidelity” denotes the need to administer treatment personalization while adhering to research-supported protocols (18, 19). Research examining the relationship between treatment integrity (i.e., therapist adherence to protocol and therapist competence) and youth outcomes has demonstrated no link between rigid treatment fidelity and outcomes (20–22). In a recent study examining 51 youth who completed individualized CBT for anxiety, therapist adherence did not influence youth outcomes (23).

One guiding framework offered by Georgiadis et al. (24) identifies the “Who, What, When, Where and Why of Strategic Flexibility,” where the therapists considers (1) who warrants treatment flexibility; (2) what treatment components should be flexibly modified, (3) when flexibility is needed (4) where flexibility is needed, and (5) why flexibility should be applied. We organize our discussion along the lines of “where.” Specifically, we examine what is known about adjusting CBT for youth anxiety in schools, community mental health agencies, telehealth, online programs, and through caregivers at home. Our focus is on flexibility within fidelity across settings, but there are also adaptations that are appropriate for variations among participant youth (e.g., youth with comorbities).

### 3.1. Schools

Educational environments are a fruitful setting in which to implement a variety of interventions because they offer the opportunity to reach a broad range of youth, including individuals who may not have otherwise had access to or been knowledgeable about needed services. Interventions in school settings can provide affordable and quality services to youth who come from under-resourced communities or to youth in lower socioeconomic brackets. Further, because youth across all demographics and with a variety of risk and resilience factors attend schools, implementing interventions in this setting can offer the first line of defense in addressing youth with more severe symptomology, especially if they are initially or frequently unable to access other intervention services for a variety of reasons (e.g., income, transportation barriers, knowledge of services, etc.). Research has found that school settings are an effective environment for implementing treatment modalities that address mental health concerns, such as youth anxiety (25). Literature indicates that CBT can be successfully implemented within a school setting (26). More specifically, Coping Cat has been successfully integrated into school settings (27, 28), highlighting the flexibility of the program and its’ ability to be modified with considerations specific to the setting (see Table 1).

**TABLE 1:** Administering CBT for youth anxiety in schools.

Potential strategy	Examples of implementation
Account for factors at a macro-level	● Consider changing session length and frequency to integrate treatment into a typical school day more seamlessly ● Modify the number of examples provided in each session (e.g., using one example to which the individual will connect) to adequately cover relevant material within a session
Account for factors at a school-level	● In cases with severe symptomology, consider referring to community settings
Adopt exposures to ensure they are appropriate for a school environment	● Allow a portion of session time at the conclusion of exposure for youth to emotionally regulate before returning to class ● Select exposures in terms of time that can be dedicated to an exposure and recovery, as well as content that is appropriate for an educational environment ● Use the time between sessions for more adventurous exposure tasks and use session time to “make sense of the exposure”
Account for factors at an individual level	● Ensure administrative support: top-down support can alleviate interventionist burnout and foster an environment where issues related to implementation are taken seriously and remedied ● When selecting an interventionist, consider if staff are overburdened, trained, and capable of conducting the intervention ● Ensure that interventionists have access to necessary resources (e.g., workbook, materials for psychoeducation, referral lists, items for exposure tasks).

There are several considerations for a school setting, in part, due to administrative and contextual issues. Domitrovich et al. (29) offered a framework: consider macro-level factors (e.g., federal, state, and district policies), school-level factors (e.g., climate, resources, decision structure), and individual-level factors (e.g., characteristics of the interventionist, attitudes toward the intervention) and how each of these may interact with the success of an intervention in schools (see Table 1). For example, at a macro-level, state/district policy can influence the allocation of funding and scheduled time to implement an intervention. Typically, the Coping Cat program is designed to accommodate 50-min weekly sessions; however, in schools, it is reasonable to modify session length to fit within a typical school schedule. When assessing the manageability of adapting the Coping Cat program into schools, Mychailyszyn et al. (30) found that it was easiest to devote 30-min sessions up to twice per week, as it more closely mirrors a routine school schedule. Coping Cat allows for flexibility in the breakdown of session information such that sessions can be split into two shorter sessions per week or, if it is not feasible to have more than one session per week, interventionists can select one activity from the session plan (preferably an activity that is expected to be of greatest interest to youth), as opposed to presenting the full gamut of activities related to the session.

A school’s organizational functioning, resources, and climate (reflected by staff impressions of their workplace) merit consideration (29). One potential barrier is implementing key components of the intervention due to constraints within the school. For example, school counselors have expressed concerns related to providing the appropriate “dosage” of the intervention, depending on symptom severity (30). Additionally, school counselors have expressed concern related to conducting exposure tasks within a school. Fortunately, there are opportunities for modifications that help to adapt the Coping Cat program to a school setting (see Table 1). It is encouraged that providers in a school setting be creative when adapting key treatment components. For example, allot time (5–10 min) at the conclusion of an exposure task to allow time for a pleasant activity, facilitating the opportunity for the youth to emotionally regulate prior to returning to class. It may also be wise to schedule longer sessions for the exposure tasks (30). If adapting certain exposure tasks does not seem conducive for a school (e.g., a specific exposure task is indicated and cannot be modified), interventions can be supplemented with outside referrals; for individuals with more severe symptomatology, one can coordinate with community-based referrals (31).

An interventionist’s attitudes (e.g., self-efficacy, experience, and psychological determinants) can contribute to the success of an intervention. In general, more positive attitudes toward the intervention and greater levels of self-efficacy are associated with more successful implementation (29). It is important to foster an environment in which an interventionist feels supported by school administration for decisions about modifying the length of sessions, session content, and appropriate exposure tasks.

### 3.2. Community mental health centers

Community mental health centers (CMHCs), despite being a widespread source of treatment for youth dealing with anxiety, have historically seldom used CBT to treat youth anxiety. A possible explanation for this hesitancy involves concerns about the practical implementation of efficacious treatment for youth anxiety in community settings, as these settings are often lacking specialized training and support (32). Effectiveness studies evaluate efficacious treatments within real-world settings and are important to determine if treatments have similar levels of success outside of a controlled efficacy study. Initial effectiveness studies investigating CBT treatment of youth anxiety in CMHCs found comparable and beneficial outcomes, but these studies should be interpreted cautiously, given several limitations.

Barrington et al. studied 54 anxious youth treated with CBT and treatment as usual (TAU) in Australia, but only youth participants were randomized, not clinicians (i.e., they used experienced CBT therapists) (33). Southam-Gerow et al. (34) used full randomization in their effectiveness study of 48 youths (i.e., they used therapists employed in community clinics to emulate community settings). Despite its methodological strengths, this study had several limitations, including small sample size (like in Barrington et al.) and non-adherence within the CBT condition. Of youth in the CBT condition, only 52% attended a full 16 sessions, and only 59% participated in exposures, both of which are known to be important and necessary for fidelity and youth outcomes (11, 35, 36) (see Table 2). While the authors noted that some of these limitations could be conceived as a byproduct of how community settings often function, and the CBT outcomes appeared to be comparable to prior efficacy trials, increased fidelity in community settings will inform how to best deliver treatment.

**TABLE 2:** Administering CBT for youth anxiety in CMHCs.

Potential strategy	Examples of implementation
Establish a baseline of training, followed by ongoing opportunities to further develop skills	● Implement an initial training (∼2 days) to help therapists hit the ground running ● Offer intermittent additional training/supervision at the therapist’s discretion to allow for/facilitate improvement
Ensure adherence to protocol (e.g., in-session exposures)	● Maintain strict expectations with regard to the number of sessions and in-session exposures. Flexibility is allowed, but minimum numbers of sessions and exposures are prioritized to preserve fidelity. Make this a core feature of the culture of the clinic! ● Regularly assess therapists to maintain accountability
Support therapists on an individual level	● Provide therapists with individual supervision for cases and as needed

A recent effectiveness study (37) exemplified fidelity: a study of 165 anxious youths conducted in CMHCs throughout Norway compared individual CBT (iCBT) and group CBT (gCBT) with a waitlist condition and found similar treatment responses in iCBT and gCBT conditions compared to previous efficacy trials. These findings show that CBT can be effectively delivered by non-specialist therapists lacking previous CBT training. Other research supports this notion of effectiveness, including a 2021 meta-analysis of 58 studies that showed CBT for the treatment of anxious youth in routine clinical care settings had similar outcomes to efficacy studies (38) and a 2020 meta-analysis of 75 studies that underscored the importance of using in-session exposure tasks in community settings (15). With the evidence that CBT of anxiety can be effective when delivered properly, it is imperative that fidelity is maintained in community settings. For instance, ensuring that youth receiving CBT for anxiety meet for the minimum number of sessions and partake in enough exposure tasks (see Table 2).

Research has identified a variety of barriers to fidelity/adherence, which can guide CMHCs’ efforts. Organizational factors influence adherence to evidence-based treatments (39)—one way to address this is by the organization-level implementation of robust initial training, intermittent “booster” trainings, and ongoing assessments of adherence. Even for therapists with little to no experience with CBT, a 2-day workshop and optional boosters provide appropriate scaffolding as a beginning (see Table 2). Another potential barrier to adherence identified by research is the perceived complexity of and variability of clients and presentations. Specific guidance and individualized supervision when implementing CBT can help ensure that each case is treated with personalized flexibility (35, 40) (see Table 2).

Related, there has been several efforts to implement CBT for youth anxiety through pediatric primary care providers (PCPs). PCPs frequently screen for anxiety—in 2022, the U.S. Preventive Services Task Force released recommendations for primary care physicians to conduct anxiety screening in asymptomatic youth from ages 8 to 18 (41). A study examining barriers to implementing psychosocial treatment in primary care found that PCPs ranked the following characteristics of treatments most important: time to employ, applicability to multiple disorders, ease of use, and ease of learning (42). In a pilot study investigating a brief CBT-based anxiety intervention delivered in a behavioral healthcare setting to 25 parent-child dyads (1–4 sessions, 15–30 min in length), pediatricians found the intervention to be feasible to administer, and parents reported reduced child anxiety (43).

### 3.3. Telehealth

Prior to 2020, there were several self-guided and therapist-mediated online computerized CBT programs that had been shown to be effective, but they did not offer the same amount of therapist contact as clinic-based CBT. Before the COVID-19 pandemic, telehealth only accounted for a very small proportion of mental health services (44). However, COVID-19 necessitated clinician use of telehealth to provide access to treatment during the global pandemic. This, in turn, generated clinician creativity in modifying treatment to the telehealth format.

Telehealth is here to stay, as 6 months into the pandemic, 90% of surveyed clinical psychologists anticipated they would continue to use telehealth after the pandemic was over (44, 45). Although there have been limited studies investigating telehealth outcomes of CBT for anxiety in youth, among adults, CBT for anxiety has been found to be similarly effective through telehealth compared to in person (46). Additionally, the therapist-client alliance is an important factor in telehealth. In a study comparing CBT for youth anxiety in a telehealth format compared to an in-person clinic-based format, youth reported no difference in the strength of their therapeutic relationship; however, parents reported slightly higher working alliance with in-person therapy (47).

Our experience conducting CBT for youth anxiety through telehealth during the COVID-19 pandemic has identified strategies to combat technical difficulties, maintain youth attention during sessions, as well as uncover some advantages provided by telehealth (see Table 3). In terms of technical matters, we’ve found that it is best for the client to have sound, video, and their face on the video screen. We also have a plan for what to do if one experiences technical difficulties sorted out ahead of time. We’ve also found that frequent distractions are the rule rather than the exception; a plan to keep distractions—both physical and virtual—to a minimum is best sorted out with the child and caregiver ahead of the session.

**TABLE 3:** Administering CBT for youth anxiety through telehealth.

Potential strategy	Examples of implementation
Implementing exposures	● Agree to clear guidelines with youth ahead of time about staying in an exposure task and not quitting or turning off the camera, even when they are experiencing anxiety ● Take advantage of the youth’s naturalistic setting by having them complete exposures at home (or in settings that are distressing to them) ● Complete exposures “on the move” and in the community (e.g., in the grocery store, on the bus) with a therapist on the phone with them
Technical matters	● Do a trial run with the youth and their caregiver ahead of the session to make sure their device and videoconferencing software is set up correctly. ● Make a plan ahead of time for how you will handle any technical issues. Will you cancel the session? Will you take 10 min to restart your computer or find a new location? Will you hold the session *via* phone?
Limit distractions	● Set up a quiet, private space appropriate for therapy. If this is not possible, ensure others who are sharing the room can use headphones or other noise-blocking devices. ● Set boundaries so that siblings and other family members do not interrupt ● Work with caregivers to set ground rules that they should not be using their phones, on social media, or on any other website during the telehealth session.
Special considerations for social anxiety/selective mutism	● Consider how they might be able to interact with peers in ways that are by technology and ways that involve in-person contact ● Consider using group-based CBT that encourages exposures with peers

One of the biggest adjustments in conducting CBT for child anxiety through telehealth is adapting the in-session exposure tasks. Telehealth can provide the opportunity to conduct an exposure within a naturalistic context. For example, the youth bring an electronic device into the real-world exposure setting. This gives a “window into the patient’s own world” (48) and may facilitate the generalizability of the benefit of the exposure. Not only can therapists conduct exposures in a youth’s own home, but a therapist can also consider being on the phone with a client as they conduct an exposure in their community.

Treating youth with social anxiety disorder (SAD) and selective mutism (SM) benefits from special considerations, such as providing opportunities to practice socializing. However, CBT has been successfully delivered in telehealth group-based formats for individuals with SAD and SM (49) (see Table 3). Telehealth can be used to provide CBT in a group format with a group of youth. Although telehealth allows for easier access to groups of individuals with similar difficulties, it also makes it potentially more difficult to complete in-person exposures.

### 3.4. Online/computerized treatment programs

There has been a flourishing of computer- and Internet-based therapy programs geared toward youth. Existing programs that deliver CBT for youth range widely with regard to the platform (e.g., from CD-ROMs to mobile applications), targeted age range (e.g., from preschool through adolescence), degree of customization possible (e.g., in terms of content, pacing, duration), and degree of therapist monitoring and contact (e.g., from “computer-based” programs with no therapist involvement to “computer-assisted” programs with abbreviated, regularly scheduled live sessions). All of these programs have the potential to increase the accessibility of quality mental healthcare for youth while circumventing or reducing common barriers to receiving evidence-based mental health treatment in traditional settings [e.g., limited availability of therapists with training in empirically supported models, therapist and client time constraints, lack of insurance or inability to afford high fees, lack of transportation and/or childcare, the stigma associated with seeing a therapist; (50)]. These benefits may be particularly salient for historically underserved populations of youth, including low-income youth, youth with marginalized identities, and youth residing in rural areas. When possible, we recommend a high degree of customization and flexibility in the mode and frequency of therapist contact to suit the needs of a greater number of youth and families (see Table 4 for specific suggestions).

**TABLE 4:** Administering CBT for youth anxiety through online computer programs.

Potential strategy	Examples of implementation
Tailor caregiver-focused sessions and check-ins to increase family engagement and ease of transfer of “coach” role from therapist to caregiver at the conclusion of the program	● Allow for flexibility in the focus of caregiver-focused session content depending on caregiver endorsement of particular cognitive and emotional responses to the youth’s anxiety symptoms, relevancy of particular parenting skills (e.g., validation, labeled praise), and the degree to which the youth needs assistance in planning and implementing exposure tasks ● Encourage caregivers to track their accommodating behaviors week-to-week; titrate therapist check-ins as needed to accomplish the goal of gradually reducing these accommodations over time
Allow flexibility in the degree of access to a therapist depending on needs for progress monitoring, increasing participant motivation, and troubleshooting skill application or hierarchy development	● Encourage therapists to provide individualized encouragement and feedback *via* online or email messages following the completion of session content ● Require that therapists regularly connect with the family *via* phone calls for abbreviated sessions/check-ins to provide encouragement, answer questions, and assess/address difficulties
Enable individualization and customization of program material based on participant goals	● Allow participants to view different examples and case studies based on common areas of fear and worry (e.g., social anxiety, health anxiety, specific phobia) ● Enable interactive hierarchy-building capabilities, in which the participant can insert and edit their own exposure plans and subjective units of distress (SUDs) ratings
Incorporate transdiagnostic skills and examples that address overlapping symptoms or underlying mechanisms	● For skills related to catching negative self-talk and cognitive restructuring, include examples using both anxious and depressive cognition ● For problem-solving skills, include tips related to behavioral activation when experiencing depressed mood and distress tolerance when experiencing emotion dysregulation ● Include information on the effects of sleep, nutrition, and physical activity on anxiety and mood symptoms
Add considerations (“quick tips”) for caregivers of youth with common co-occurring neurodevelopmental or psychological concerns	● For caregivers of youth with ADHD, suggest strategies to help youth learn and generalize content (e.g., repetition, use of visual stimuli, reducing distractions when completing session content) ● For caregivers of youth with autism spectrum disorder, suggest increasing parental involvement, potentially slowing the pace, and incorporating the youth’s preferred interests ● For caregivers of youth with obsessive-compulsive disorder, suggest emphasizing exposure and response prevention and deemphasizing cognitive restructuring
Allow for flexibility in treatment pacing and duration	● Enable the ability to complete sequential session content at varying intervals to increase convenience for families with shifting schedules ● Offer follow-up booster sessions or check-ins for participants who may benefit from support with applying skills to new situations and contexts
Vary graphics and interactive activities by age to increase appeal	● Incorporate a mix of text, audio, video, and interactive activities suitable for different ages (children; adolescents) ● For children, gamify session content such as by having them earn points for the completion of session content that enables them to “level up” ● For adolescents, ensure that graphics and materials are engaging (e.g., use of relevant examples, not text-dense) and not overly childish
Build program on a user-friendly platform that is accessible *via* multiple device types, and that allows for real-time exposure tracking and recording capabilities	● Build an application that is accessible *via* mobile phone and desktop computer to enable accessibility regardless of participant location ● Include capability for participants to input minute-to-minute SUDs ratings, automatically generating a graph depicting change over time ● Include capability to input exposures, which may be utilized by therapists for troubleshooting purposes, or the family to review in future weeks

Another potential benefit of computerization is greater assurance of fidelity and adherence compared to live therapy, given that at least a portion of online and computer-based program content is systematically delivered (51, 52). However, concerns have been raised regarding the ability of these standardized programs to flexibly address the complex needs of youth and families. We propose ways for online CBT interventions for youth anxiety to facilitate greater flexibility and thus more effectively serve the needs of diverse youth and families. Considerations for increasing flexibility begin when developing program design and capabilities and continue through to roles assigned to therapists or “coaches” (see Table 4). We caution online intervention developers to remain vigilant about issues of fidelity by ensuring that core treatment elements are incorporated in some manner for all participants and, accordingly, encourage incorporating “check-ins” on participants’ experiences throughout the program.

One computer-assisted program for youth with anxiety ages 7–13 is Camp Cope-A-Lot [CCAL; (53)] based on the Coping Cat treatment program (28, 53). The development team included researchers, child psychologists, programmers, and graphic designers, and worked with the goal of creating a program that may be used in settings such as private practices, CMHC, schools, and ultimately in youths’ homes with caregiver assistance (53). Parent input was incorporated in the design of the intervention as well. CCAL is experiential, interactive, and designed to engage youth in middle childhood and early adolescence. In the context of prior research, CCAL included assistance from mental health providers who served as “coaches” for participants, with responsibilities including symptom monitoring, encouraging compliance, and maintaining the integrity of core CBT program elements (51). Of note, several opportunities for flexibility were built into CCAL, including participant ability to customize their own exposure tasks, move through program content at their own pace, and choose between options of theme music and video game rewards. A randomized clinical trial (51) found that both in-person and computer-delivered treatment was effective; there were no statistical differences in gains among youth assigned to CCAL versus those assigned to individual CBT delivered in a traditional manner at both posttreatment and follow-up time points. Both interventions outperformed a computer-assisted education, support, and attention condition. CCAL, as well as Child Anxiety Tales, a web-based program for parents, are now available as online programs (54).

### 3.5. At-home

Traditionally, much work in youth anxiety is done in the therapist’s office, but both experience and data attest to the importance of generalizing to the home environment. Participation in and compliance with treatment is best when it continues after leaving the clinic. It is encouraged (e.g., *via* homework) that children and adolescents continue to work toward treatment goals at home, often with the help of caregivers or others who live with them, for example, through the elimination of accommodations and by practicing exposures.

Accommodations in the home are usually done by caregivers, though others living in the house could also contribute. A common form of accommodation in the home reported by caregivers is changing the child’s or the family’s schedules, routines, or expectations to avoid the child’s discomfort and avoid a possible emotional/behavioral outburst. In a study of 313 parents seeking treatment for their child’s anxiety, it was found that parents who believe accommodation helps maintain behavioral/emotional control accommodate more (55). A study of 73 youths (ages 7–12) quantified these results, showing that reduced accommodation predicted progress in the child overcoming anxiety (56). There are treatments designed specifically for parents that focus on eliminating accommodation in the home (57–59) that were found to be comparably efficacious as individual CBT. Table 5 includes examples of ways caregivers could reduce accommodation in the home. We encourage recommending a reduction or elimination of accommodations in the home to allow for increased mastery or the development of confidence in the child’s abilities to tackle new and challenging situations on their own.

**TABLE 5:** Administering CBT for youth anxiety with caregivers.

Potential strategy	Examples of implementation
Provide resources for caregivers to address their own maladaptive behavior or cognition	● If caregiver psychopathology is a factor in the child’s anxiety or treatment, offer referrals and/or options for their own work ● Demonstrate concrete, relevant, and detailed examples of positive modeling that caregivers can readily implement at home. ● Provide parent-focused session to teach about exposure therapy and provide examples of possible exposures to try at home. ● Provide parent-focused session to teach about accommodation and provide support for removing accommodations through suggestions for rewarding brave behavior.

Some anxious youth may experience less anxiety in the home, where they feel safe and comfortable. This experience is often the case with social anxiety and certain phobias. However, there are also children who experience a great deal of anxiety in the home (e.g., worries about bedtime for those who struggle with separation anxiety or a fear of the dark, worries about home invasions). Exposure tasks are one way therapists generalize treatment to other settings, such as the home. Exposures at home, much like in any other environment, are critical to treating anxiety, and caregiver involvement is necessary for effective youth participation (60). However, parents may want to maintain their role as parents and rely on a professional for treatment (61), creating an obstacle for generalizing treatment to the home. A session dedicated to parental psychoeducation about exposures and how best to implement an exposure at home is helpful (see Table 5) in ensuring treatment is generalized to the home (62).

## Author contributions

PK and JN contributed to the conceptualization. All authors contributed to the writing, editing, and approval of the submitted version.
